# Integrated bioinformatics analysis identifies the effects of Sema3A/NRP1 signaling in oligodendrocytes after spinal cord injury in rats

**DOI:** 10.7717/peerj.13856

**Published:** 2022-08-16

**Authors:** Rong Hu, Mengting Shi, Haipeng Xu, Xingying Wu, Kelin He, Yi Chen, Lei Wu, Ruijie Ma

**Affiliations:** 1 Zhejiang Chinese Medical University, Key Laboratory of Acupuncture and Neurology of Zhejiang Province, Third School of Clinical Medicine (School of Rehabilitation Medicine), HangZhou, China; 2 Department of Acupuncture and Moxibustion, The Third Affiliated Hospital of Zhejiang Chinese Medical University (Zhongshan Hospital of Zhejiang Province), HangZhou, China

**Keywords:** Bioinformatic analysis, Spinal cord injury, Oligodendrocyte, Sema3A/NRP1signal, PDGFRα

## Abstract

**Objective:**

To investigate the effect of Sema3A/NRP1 signaling in oligodendrocytes (OLs) after spinal cord injury.

**Methods:**

Three analysis strategies, namely differential expression gene analysis, Gene Ontology (GO) enrichment, and Kyoto Encyclopedia of Genes and Genomes (KEGG) pathway analysis, were applied. The protein-protein interaction (PPI) network was constructed using the STRING website to explore the correlation between Sema3A/NRP1 and oligodendrocytes. Then, the T10 spinal cord segment of rats was injured by the Allen method to establish a spinal cord injury (SCI) model. Real-time quantitative PCR, Western blotting, Nissl staining and immunofluorescence staining were used to detect the effect of Sema3A/NRP1 signaling on oligodendrocytes *in vivo*.

**Results:**

After the SCI model was established, significantly fewer oligodendrocytes were observed. At the same time, R software was used to analyze the expression of related genes, and NRP1 expression was increased. PCR also demonstrated similar results, and NRP1 ligand Sema3A was also upregulated. KEGG and GO functional enrichment analysis indicated that the SCI model was mainly related to cytokine interaction, cell proliferation, differentiation and maturation. Interestingly, we found that NRP1 was involved in semaphorin-plexin signaling pathway neuronal projection guidance and axon guidance, mediating cell growth and migration. Moreover, Sema3A/NRP1 signaling was closely associated with platelet-derived growth factor receptor *α* (PDGFR*α*) in the PPI network. When Sema3A/NRP1 signaling was specifically blocked at early stages, PDGFR*α* expression was effectively inhibited, and the expression of OLs was promoted. Furthermore, inhibition of Sema3A/NRP1 signaling increased the Basso-Beattie-Bresnahan (BBB) score of lower limb motor function in SCI rats and promoted the survival of motor neurons in the ventral horn of the injured spinal cord.

**Conclusion:**

Our data suggest that Sema3A/NRP1 signaling may regulate the development of OPCs and OLs after SCI, thereby affecting functional recovery.

## Introduction

Spinal cord injury (SCI) has become a major public health concern with no effective treatment. Clinically, SCI is mainly manifested by irreversible neurological impairment, resulting in a series of dysfunctions (Almeida et al., 2018). Current evidence suggests that there are approximately 133,000 to 226,000 new cases of SCI per year globally. In the United States, the hospital charges related to SCI are close to US$9.7 million per year, which burdens the medical care system, the patient’s family and society (Wyndaele & Wyndaele, 2006; Kumar et al., 2018). To date, little is currently known about the underlying mechanisms, warranting further studies.

It is widely acknowledged that oligodendrocyte precursor cells (OPCs) and oligodendrocytes (OLs) are widely distributed in the central nervous system (CNS). OLs are the glial cells of myelin axons derived from OPCs, playing an important role in the integrity of axons, maintaining the normal structure of axons and optimizing axon conduction (Gritsch et al., 2014; Welsh & Kuenas, 2018). SCI and its secondary impairment may lead to loss of OLs and neurological dysfunction (Baumann & Pham-Dinh, 2001). Therefore, the development and recovery of OL are essential during the recovery process after nerve damage.

Neuropilin1 (NRP1) is a transmembrane glycoprotein mainly expressed in dendrites and axons of neurons that plays a key role in the functional recovery of SCI after extracellular binding with Semaphorin3A (Sema3A)  (Parker et al., 2012). In recent years (Shim et al., 2013; Nakanishi, Fujita & Yamashita, 2019), the role of Sema3A/NRP1 signaling in directional migration of OLs and axonal regeneration after nerve injury has been reported. In addition, Sema3A/NRP1 signaling is a directional guide for oligodendrocyte migration in the hallmark of multiple sclerosis (Jiang et al., 2010). However, the role of Sema3A/NRP1 signaling in regulating OLs development after SCI remains largely unknown. This study observed the effects of Sema3A/NRP1 signaling in OLs associated with SCI in rats to explore the molecular mechanism underlying functional recovery.

## Materials and Methods

### Analysis method of the data spectrum

#### Data download and processing

The gene expression data for the present study was downloaded from Gene Expression Omnibus (GEO) database (https://www.ncbi.nlm.nih.gov/geo/) using the accession number (Chamankhah et al., 2013). The dataset GSE450006 was based on the GPL1355 platform and contained 4 spinal cord samples from sham rats and 20 from SCI model female rats. 24 samples were selected for further data processing. Specifically, the raw expression datasets were log2-transformed for further analysis. Subsequently, according to platform annotation files, the probes were matched with corresponding genes, and non-matched probes to gene symbol were removed. When multiple probe names were assigned to a single gene symbol, the values were averaged. Finally, the data was assembled for subsequent analysis.

#### Identification of DEGs

Data were collated as previously described in Ren et al. (2020). Specifically, R software and related R packages were used to normalize and analyze differentially expressed genes (DEGs). Log2 fold change (FC) greater than 2 and adjusted *p*-value < 0.05 were selected as the cutoff criterion for DEGs screening. The analysis results were presented by heat maps and volcano plots drawn in R Studio software (version:1.2.1335; RStudio Team, 2015).

#### Functional enrichment analysis

To further analyze the biological processes of DEGs in the sham group compared with the SCI group, Gene Ontology (GO) and Kyoto Encyclopedia of Genes and Genomes (KEGG) pathway enrichment analyses were carried out using the cluster Profiler in R package and R studio software (Yu et al., 2012). Significantly enriched GO terms and KEGG pathways were screened using the criteria: *p*-value <0.05 and an enriched gene count >2.

#### Protein-protein interaction (PPI) network analysis

To explore the mutual relationship between proteins encoded by different genes, DEGs were imported into the STRING website (version:11.0) for further analysis (Szklarczyk et al., 2019; Snel et al., 2000; Athanasios et al., 2017). The lowest interaction score was greater than 0.4, and isolated nodes in the network were removed. Then the analysis results were output to the TSV format file, and the detailed processing and module analysis were carried out by Cytoscape software (version:3.7.1). Molecular Complex Detection (MCODE)  (Fischer et al., 2015) is a plug-in downloaded from Cytoscape App Store used to find closely connected nodes in a complex network based on topology. In the present study, we applied plug-in MCODE to select critical modules of PPI network.

### Animals

All healthy male SD rats (eight-week old, 220 ± 10 g) were provided by Shanghai Xipu Bikai Shanghai Xipu Laboratory Animal Co. (animal license No: SCXk (Shanghai) 2018-0006) and were housed in the Experimental Animal Center of Zhejiang Chinese Medical University (AAALAC, animal license No: SYXK (Zhejiang) 2018-0012). The animals were housed in environmentally-controlled standard conditions (illumination, 12/12 h light/dark cycle; humidity, 50–60%; room temperature, 21–23 °C) with ad libitum access to food and water. 3% pentobarbital sodium (0.15 ml/kg, i.p.) was intraperitoneally injected before the end of the experiment. Rats were divided into a sham group (sham, only underwent laminectomy does not damage the spinal cord), the SCI model group (SCI, underwent laminectomy and damage the T10 spinal cord, details in ‘SCI Rat Model Establishment’), the SCI+AAV Sema3A group (spinal cord stereotactic injection of AAV virus, details in 2.4), and the SCI+AAV NC group (spinal cord stereotactic injection of AAV control virus, details in ‘Virus Injection’). All experiments were performed in compliance with relevant ethical regulations and approved by the animal ethics committee of Zhejiang Chinese Medical University (IACUC-20190128-02). All the experimental protocols strictly followed the National Institutes of Health guidelines for the care and use of laboratory animals (NIH Publication No. 8023).

### SCI rat model establishment

To establish a contusive SCI model at T_10_, contusive injury was induced on the T_10_ spinal cord using the MASCIS weight-drop device with a 5 × 10 g/cm gravitational potential energy after T_10_ laminectomy. The severity and consistency of injury were verified by checking the bruise on the spinal cord or tail-flick of the rats after weight drop. All animals received intraperitoneal injections of penicillin (100 U/d) for three days to prevent infection.

### Virus injection

To further verify the effect of Sema3A/NRP1 signaling after SCI, we designed an SNCA-shRNA sequence plasmid and integrated it into adeno-associated virus (AAV), as previously described (Hu et al., 2020), and injected it into rats to downregulate Sema3A expression. Briefly, at 21 days before modeling, 0.5 ul AAV2/9-U6-shRNA(Sema3A)-CAG-tdtomato or a negative control AAV2/9-U6-shRNA(luciferase)-CAG-td tomato virus (Shanghai Taitool Bioscience Co., Ltd.) were injected bilaterally between T9 and T10 spinal cord using a 10 ul Hamilton syringe after rats anesthetized with pentobarbital sodium (40 mg/kg, i.p.). Vessels and nerves were avoided while injecting the virus. The viral titer was 7.5 × 10^12^ vg/ml. 7 × 10^9^ vg virus was given to every animal.

### Nissl staining

Procedures for Nissl staining were as follows. Briefly, after dehydration in 30% sugar solution, the tissues were sliced with a freezing microtome. The slices were incubated with nissl staining solution in a 37 °C constant temperature water bath for 10 min, followed by alcohol gradient decolorization for 1 min each, and washed with distilled water for 30 s immediately. Finally, the slices were sealed with neutral resin. Images were taken with a light microscope.

### Real-time quantitative PCR (qPCR) analysis

First, total RNA was extracted from the T10 spinal cord tissue and the quality of RNA tested (Invitrogen, Carlsbad, CA, USA), cDNA was synthesized using PrimeScript RT reagent Kit (TaKaRa). qRT-PCR was conducted by using the Fast Start Universal SYBR Green Master kit (TaKaRa Bio Inc, Beijing, China) with a 20 µl reaction system. The data for qRT-PCR were collected with CFX96 Real-Time System (BioRad, USA). All qRT-PCR results were analyzed using comparative Ct methods (2 − ΔΔCt). Primer sequences are listed in Table 1.

**Table 1:** The primers used in qPCR.

Primers	Forward	Reverse	Amplicon size (bp)
Sema3A	TGGAACTGCTGCGGATTTCATGG	AGTCGTGCTGCTCGGTCCTG	89
NRP1	GGCGACAAGAACATCTCCAGGAAG	AACAGGCACAGTACAGCACAACTC	136
Olig2	CCAAGATCGCCACGCTGCTG	TCGCTCACCAGTCTCTTCATCTCC	85
Sox10	CGAGGCAGACGATGACAAGTTCC	CTCTTGCTGGCACCGTTGACC	117
PDGFR*α*	GTGCCGCTGAGTTCGTCCTTC	GCTGAGGCGTTGACCACTTCC	161
*β*-actin	TGTCACCAACTGGGACGATA	GGGGTGTTGAAGGTCTCAAA	165

### Western blotting

Rat spinal cord tissue was homogenized with radioimmunoprecipitation assay (RIPA) buffer. Protein concentrations were determined using the bicinchoninic acid (BCA) assay. Furthermore, equal quantities of protein were separated *via* 8% SDS-PAGE gel and transferred by electroblotting polyvinyl difluoride (PVDF) membranes. The membranes were blocked with 5% nonfat milk in TBST containing 0.1% Tween 20 at room temperature for 1.5 h and then incubated overnight at 4 °C with primary antibody: semaphorin3A (GTX37550, 1:500), neuropilin1 (ab81321, 1:1,000), Platelet-derived growth factor receptor *α* (PDGFR *α*) (ab203491, 1:1,000), and *β*-actin (ab20271, 1:5,000). The following day, the membrane was incubated at room temperature for 2 h with secondary antibodies (CST7074s, 1:2000). Finally, immunoreactive bands were detected by enhanced chemiluminescence and visualized with an Image Quant LAS 4000. For statistical comparisons, primary antibody values were normalized against *β*-actin for each run in the normal Western blots. The density of each band was measured by ImageJ analysis software.

### Immunofluorescence staining

Frozen sections were used for immunofluorescence. Transverse spinal cord sections (25 µm) were cut on a frozen microtome and installed on gelatin-coated glass slides. Then, the tissues were incubated overnight with the following antibodies at 4 °C: semaphorin3A (GTX37550, 1:200), neuropilin1 (AF566, 1:50), PDGFR *α* (ab203491, 1:500). The signal was detected with the corresponding second antibodies conjugated to Goat anti-rabbit Alexa Fluor-488 (111-545-144, 1:600), Donkey anti-rabbit Alexa Fluor 647 (711-605-152, 1:600) or Donkey anti-goat Alexa Fluor 488 (ab150129, 1:600) and viewed by Nikon A1R laser scanning confocal microscope.

### Behavioral testing

Hindlimb locomotor function was evaluated using Basso-Beattie-Bresnahan (BBB) score. As previously described (Wei et al., 2017), the BBB scale ranges from 0–21 and reflects the extent of hind limb movements , weight support, stepping ability, coordination, and trunk stability. Evaluation and analysis were conducted by a specialist with an independent assistant blinded to the group allocation. Then the motor performance of the rats in an open field was scored, including hind limb joint movement, weight support, plantar stepping, coordination, paw position, and trunk and tail control. Scoring was performed as illustrated in Table 2.

**Table 2:** Basso Beattie Bresnahan locomotor rating score.

Score	The ability of Lower limb motor
0	There is no visible hindlimb (HL) movement
1	Light movement of one or both joints, usually hip and/or knee
2	Broad movement of one joint or joint and slight movement of the other
3	Extensive movement of the two joints
4	Light movement of three joints
5	Light movement of two joints and wide movement of the third
6	Broad movement of the two joints and light movement of the third
7	The extensive movement of all three joints of HL
8	The ball of the foot without weight support or without weight support
9	The soles of the feet occasionally bear the weight of the ground (for example, when stationary), frequent or consistent load-bearing movements of the dorsal claw, without the soles of the feet supporting the movement
10	Paw surface occasionally moves with load bearing without FL-HL coordination
11	Paw surface has more load bearing movement and no FL-HL coordination
12	More load bearing movement and occasional FL-HL coordination on paw surface
13	Common paw bearing movement and frequent FL-HL coordination
14	Continuous palm-surface bearing movement with consistent FL-HL coordination, or common palm-surface movement, continuous fore-hind limb coordination, and occasionally dorsal claw movement
15	Continuous paw and palm bearing movement and consistent FL-HL coordination, no or occasional ground grasping movement in the forward motion of the forelimbs, and the position of the main claw parallel to the body at the initial contact
16	In the gait, the continuous paw landing and the coordinated movement of the front and rear limbs are common in the process of grasping the ground; The main claw position is parallel to the body at initial contact, and rotates after load transfer
17	In the gait, the continuous paw landing and the coordinated movement of the front and rear limbs are common in the process of grasping the ground; The main claw position is parallel to the body at initial contact and load transfer
18	In the gait, the continuous paw touches the ground in a coordinated manner with the front and rear limbs. In the process of progress, the continuous paw grasps the ground. The position of the main paw is parallel to the body at the initial contact
19	In the gait, the continuous paw touches the ground in a coordinated manner with the front and rear limbs. The continuous paw grasps the ground in the process of advancing. The position of the main paw is parallel to the body at the initial contact and load transfer
20	The position of the main claw is parallel to the body during initial contact and weight transfer. The trunk is unstable and the tail kept cocking up
21	The position of the main claw is parallel to the body at the initial contact and load transfer, and the trunk is stable and the tail kept cocking up

### Statistical analyses

The statistical analyses between control and experimental groups were conducted by an unpaired *t*-test or one-way ANOVA followed by Tukey Kramer tests in GraphPad Prism7. Results are expressed as mean ± SEM. A *p*-value < 0.05 was statistically significant.

## Results

### The expression of OLs and PDGFR *α* after SCI

**Figure 1:**
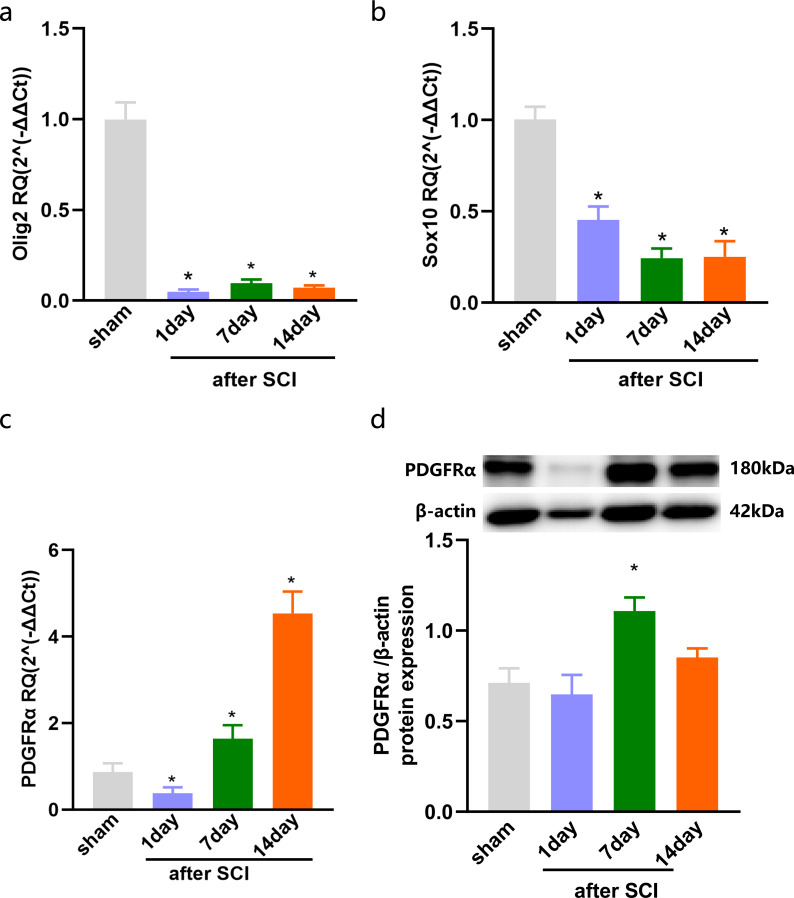
The expression of OLs and PDGFR*α* after SCI. Decreased oligodendrocytes after spinal cord injury. (A–C) qPCR validation of the Olig2 (A) Sox10 (B) and PDGFR*α* (C) expression in the spinal cord of rats after SCI. (D) PDGFR*α* protein expressions in SCI rats *vs.* sham rats. *n* = 6. Statistical significance was assessed by one-way ANOVA, *p < 0.05, Mean ± SEM.

The injured spinal cord tissues were harvested on days 1, 7, and 14 after SCI, and the expression of OLs was detected by qPCR. The results showed that compared with the sham group, olig2, the early specific marker of OLs was significantly reduced after SCI in the injured spinal cord tissue of rats (Fig. 1A). Sox10, the mature OLs specific marker, exhibited low levels after SCI (Fig. 1B). Furthermore, the specific marker of OPCs, Platelet-derived growth factor receptor *α* (PDGFR *α*), was significantly increased after SCI (Fig. 1C). We further detected the expression of PDGFR *α* in spinal cord tissue of SCI by western blotting, and the results showed that the expression was increased at 7 and 14 days (Fig. 1D). Consistent with previous studies (Assinck et al., 2017) that reported a large number of OL deaths after SCI, and the spontaneous differentiation of OPCs could be stimulated.

### Increased expression of Sema3A and NRP1 after SCI

**Figure 2:**
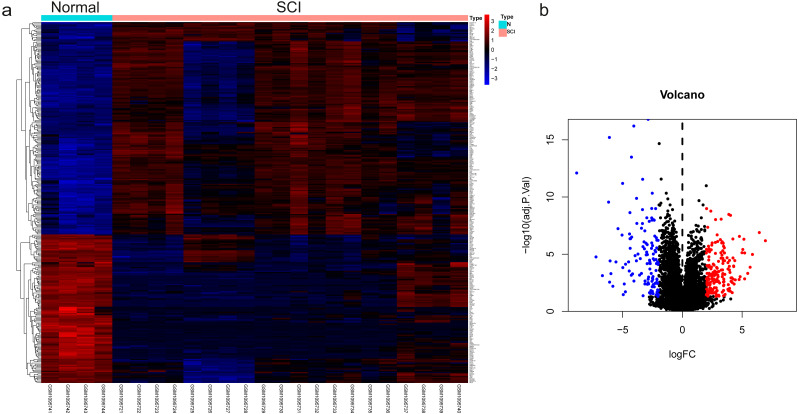
The differential genes were identified. (A) Heat map displaying the gene expression value of SCI and sham operation groups after hierarchical clustering of DEGs. (B) Volcano plot showing gene expression profiles in SCI group compared with the sham group.

**Figure 3:**
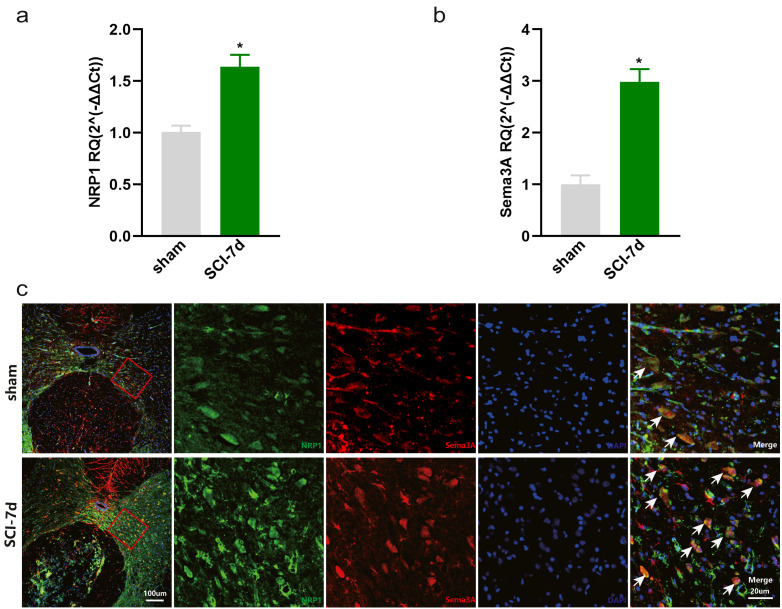
Increased expression of Sema3A and NRP1 after SCI. (A, B) qPCR validation of the upregulation of Sema3A and NRP1. *n* = 6 Statistical significance was assessed by the unpaired two-tailed Student’s *t*-test, *n* = 3. *p < 0.05, Mean ± SEM. (C) Fluorescence image of Sema3A and NRP1 binding together. Red fluorescence represents Sema3A, green fluorescence represents NRP1, and blue fluorescence represents DAPI nuclei staining.

The dataset GSE450006 based on the GPL1355 platform contained four spinal cord samples from sham rats and 20 from SCI model rats. A total of 299 DEGs were obtained, including 176 upregulated and 123 downregulated genes (Fig. 2A), and the volcano plot showed upregulated and downregulated gene expression in each sample (Fig. 2B). We observed that OLs were significantly decreased (Fig. 1) while NRP1 and Sema3A were upregulated (Figs. 3A and 3B) in SCI rats. Based on the fact that NRP1 can only exert its signal transduction function after forming a complex with Sema3A extracellularly (Parker et al., 2012; Hu et al., 2020), we performed immunofluorescence staining on the spinal cord tissues of sham and SCI seven-day rats. We observed that Sema3A and NRP1 were co-expressed in the SCI model (Fig. 3C).

### Analysis of the function and pathway of DEGs in SCI model rats

**Figure 4:**
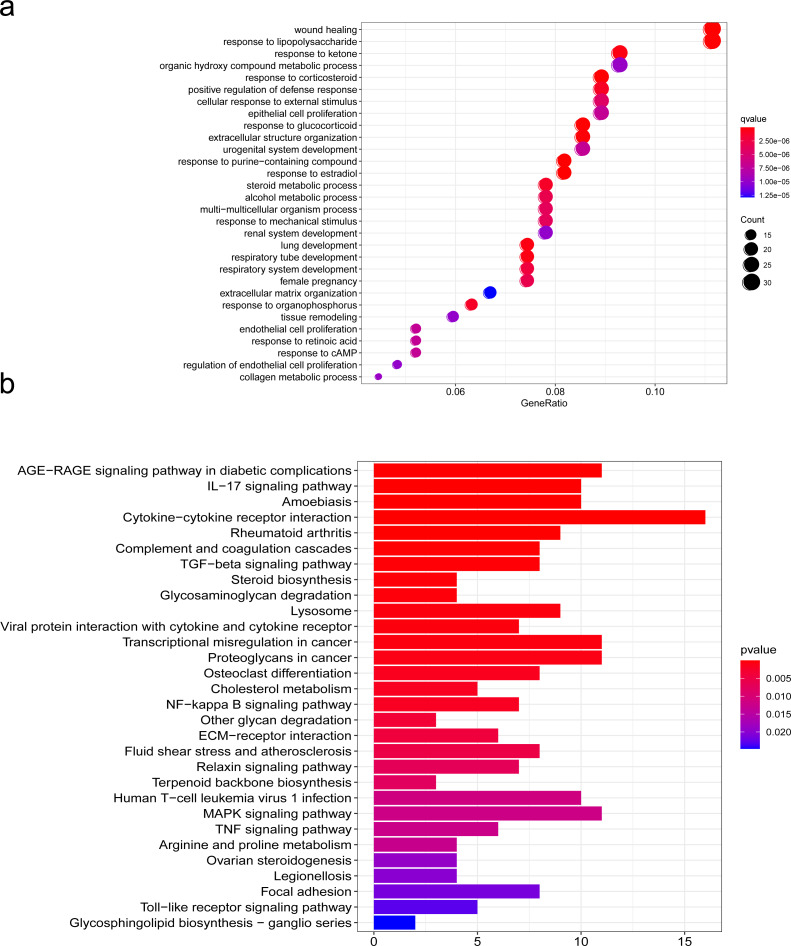
Functions analyzed by gene ontology (GO) enrichment of SCI differentially expressed genes (DEGs). GO and KEGG pathway analysis. (A) Gene Ontology (GO) enrichment analysis of differentially expressed genes (DEGs) of SCI. (B) KEGG pathway analysis of DEGs.

To further explore the mechanism of SCI, we performed GO and KEGG pathway enrichment analyses of DEGs with the cluster profiler package in R software. GO is one of the most widely used ontologies, which annotates genes in terms of their molecular functions, cellular constituent, and biological processes (Feunang et al., 2016). Moreover, the KEGG database is an important tool for identifying functional and metabolic pathways  (Sun et al., 2021). In this study, we used the R package “clusterProfiler” that automates the process of biological-term classification and the enrichment analysis of gene clusters. The screening criteria for statistically significant GO terms or pathways was a *p*-value less than 0.05. We found that the biological functions of DEGs were mainly related to wound healing, cell growth, chemotaxis and metabolism regulation, extracellulaar matrix organization in the SCI group (Fig. 4A). KEGG pathway analysis of DEGs between the SCI and the sham groups revealed significant enrichment in the interaction between cells and cytokines, cell proliferation, differentiation and maturation, ECM-receptor interaction (Fig. 4B). We further condensed all enriched pathways processed by KEGG and GO functional enrichment analysis, summarized all NRP1 involved in the results (Table S1), among which unexpected findings include the semaphorin-plexin signaling pathway involved in neuron projection guidance and axon guidance, mediating cell growth and migration (Table 3).

**Table 3:** Functions were analyzed by gene ontology (GO) enrichment of SCI differentially expressed genes (DEGs).

Ontology	ID	Description	*p*.adjust	Count
BP	GO:1901342	Regulation of vasculature development	0.000025059041	20
BP	GO:0032103	Positive regulation of response to external stimulus	0.000040991881	19
BP	GO:0001558	Regulation of cell growth	0.001189009268	15
BP	GO:0090130	Tissue migration	0.001373745350	19
BP	GO:0032102	Negative regulation of response to external stimulus	0.001546559919	15
BP	GO:0050920	Regulation of chemotaxis	0.002958470252	16
BP	GO:2001233	Regulation of apoptotic signaling pathway	0.008435229403	12
BP	GO:0045926	Negative regulation of growth	0.021059552008	11
BP	GO:0031346	Positive regulation of cell projection organization	0.038979640240	15
BP	GO:0051496	Positive regulation of stress fiber assembly	0.054125841048	4
BP	GO:0031032	Actomyosin structure organization	0.056226885639	8
BP	GO:1902287	Semaphorin-plexin signaling pathway involved in axon guidance	0.061284157525	2
BP	GO:0008045	Motor neuron axon guidance	0.063754433336	3
MF	GO:0005539	Glycosaminoglycan binding	0.000006050609	17
MF	GO:0008047	Enzyme activator activity	0.022590621407	15

### Sema3A regulates the expression of NRP1 after SCI

**Figure 5:**
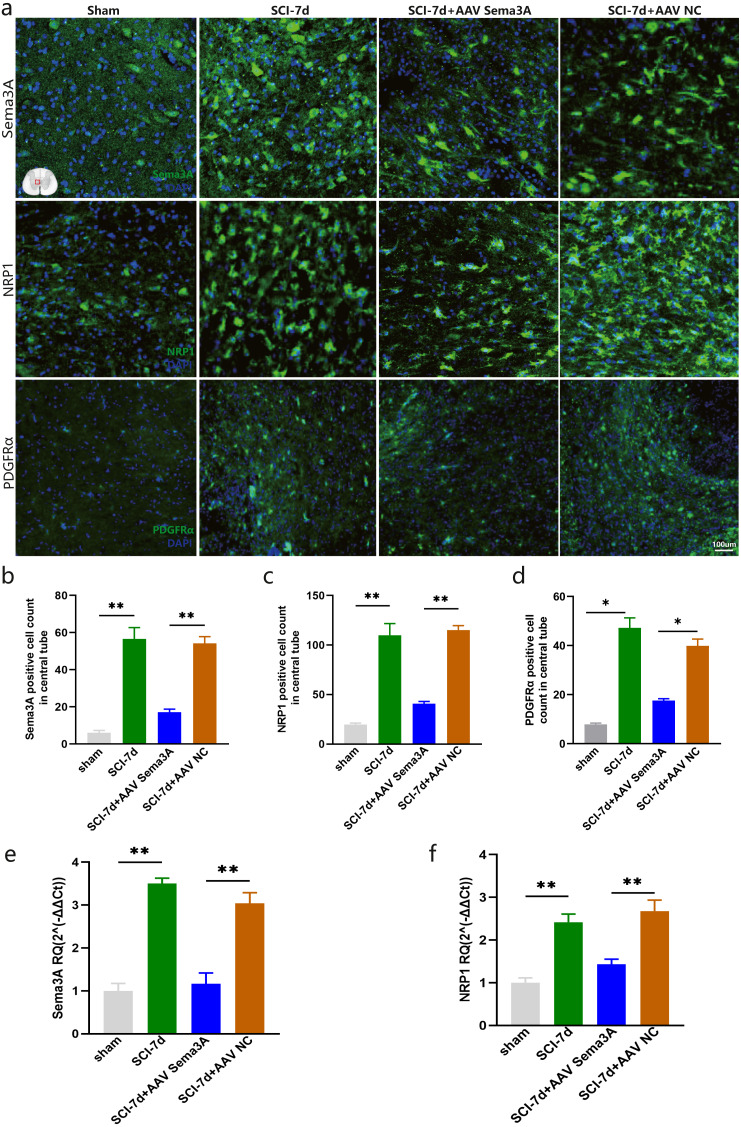
Sema3A knockdown downregulated the expression of NRP1 and PDGFR*α* after SCI. (A) A representative diagram of Sema3A and its receptor NRP1 expression after SCI in the spinal cord in the sham group, SCI-7d group, SCI-7d + AAV Sema3A group, and SCI-7d+AAV NC group was shown by immunofluorescence. (B–D) Quantification of the immunofluorescence data in (A), *n* = 3. (E–F) qPCR validation of the expression of Sema3A and NRP1. *n* = 6 rats/group. Statistical significance was assessed by one-way ANOVA, **p < 0.01, Mean ± SEM.

To further define the relationship between Sema3A and NRP1, we designed SNCA-shRNA sequence plasmids with rad fluorescent tags and integrated them into an adeno-associated virus (AAV). Subsequently, we injected AAV2/9-U6-shRNA (Sema3A)-CAG-tdtomato into the junction between T_9_ and T_10_ spinal cord rats 21 days before modeling to knock down the expression of Sema3A. Four weeks after injection, we took the thoracic spinal cord for sectioning and observed it under the microscope. It was found that the T10 segment had the best effect of AAV virus transfection (Fig. S1). Next, we observed the changes in NRP1 and Sema3A after SCI. The immunofluorescence analysis results showed that compared with the sham group, the expression of Sema3A and NRP1 in spinal cord tissue of SCI rats increased, and knockdown of Sema3A could significantly downregulate the expression of NRP1 (Figs. 5A–5C). The qPCR results were consistent with the results obtained by immunofluorescence analysis results(Figs. 5E–5F).

### Sema3A/NRP1 signaling regulates the expression of PDGFR *α* and OLs

To investigate the role of Sema3A/NRP1 signaling in SCI, we constructed the PPI network using STRING database and visualized it with Cytoscape. The upregulated genes were highlighted in red, downregulated genes in green, key genes in blue, and gray lines represented the protein interaction relationships. The results imply a possible regulatory relationship between the Sema3A/NRP1 and PDGFR *α* (Fig. S2).

**Figure 6:**
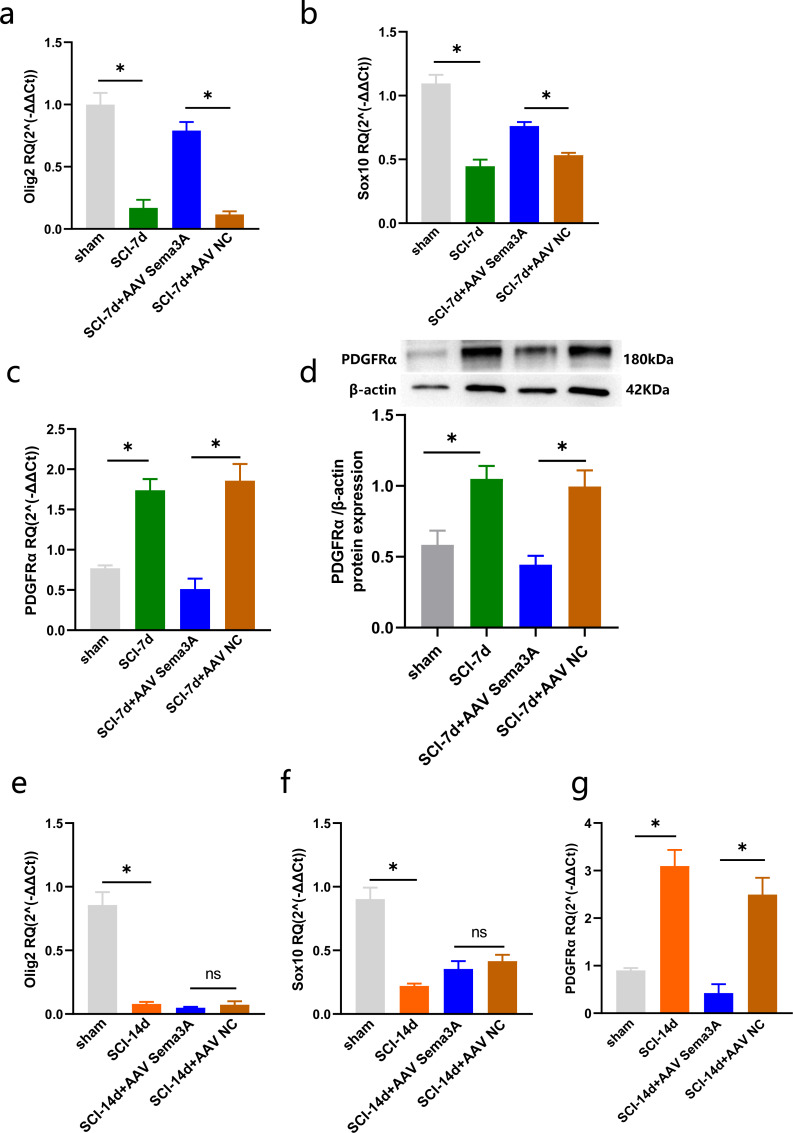
Sema3A/NRP1 regulates the expression of PDGFR*α* and Oligodendrocytes. (A–C) qPCR validation of the Olig2 (A), Sox10 (B) and PDGFR*α* (C) genes in the spinal cord of rats at 7 days after SCI. (D) PDGFR*α* expression in the spinal cord of sham, SCI-7d, SCI-7d+AAV Sema3A and SCI-7d+AAV NC groups measured by Western blot. (E–G) qPCR validation of the Olig2 (E), Sox10 (f) and PDGFR*α*(G) genes in the spinal cord of rats at 14 days after SCI. *n* = 6 rats/group. Statistical significance was assessed by one-way ANOVA, *p < 0.05, Mean ± SEM.

We measured PDGFR *α* mRNA and PDGFR *α* protein levels by real-time PCR, immunofluorescence, and western blotting. The results revealed that the overexpression of PDGFR *α* in SCI model rats was significantly reduced by inhibition of Sema3A/NRP1 signaling (Figs. 5A, 5D, 6C, 6D, and 6G). To better understand the cellular mechanism of Sema3A/NRP1signaling in mediating motor function recovery in SCI, we investigated whether Sema3A/NRP1 signaling was involved in the maturation of OLs after SCI, which is also a key link in neurological function recovery (Tetzlaff et al., 2011). The qPCR results indicated that the expression of Olig2 and Sox10 of SCI group rats (after Sema3A/NRP1 signaling inhibition) significantly increased on day seven (Figs. 6A–6B) but showed no significant change on day 14 (Figs. 6E–6F). The above data indicate that Sema3A/NRP1 signaling may regulate the expression of PDGFR *α* and OLs.

**Figure 7:**
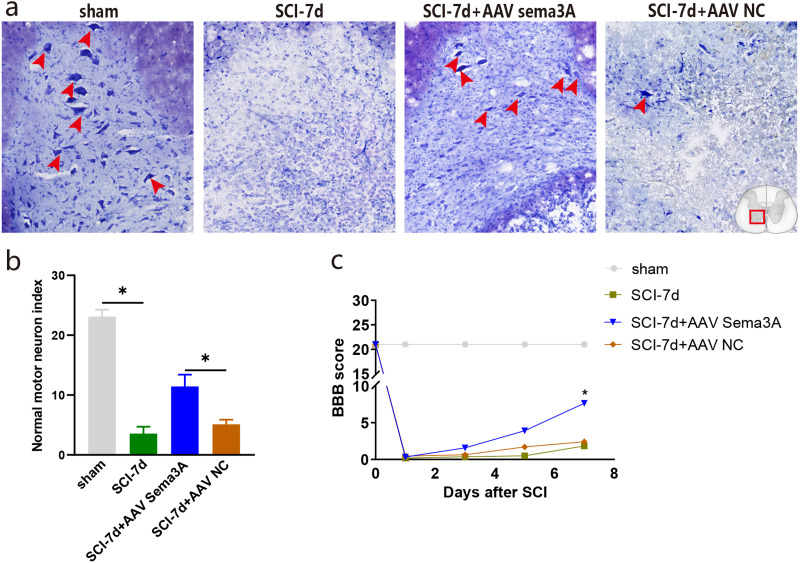
Inhibition of Sema3A/NRP1 signaling promotes neural function recovery after SCI. (A) Nissl staining of the ventral horn of the spinal cord. (B) The surviving motoneurons of the ventral horn of the spinal cord were quantified by nissl staining, *n* = 3, (C) The motor function score of each group on day 7 compared with the SCI-7d group, Statistical significance was assessed by a two-way ANOVA.

### Inhibition of Sema3A/NRP1 signaling promotes function recovery after SCI

The recovery of hind limb function was assessed using the BBB rating scale to examine the effect of Sema3A/NRP1 signaling on locomotor recovery after SCI. The line graph shows the BBB score for each group within 1 week after SCI. We found that all rats had a BBB score of 21 before SCI and lost locomotor function after SCI. When Sema3A/NRP1 signaling was inhibited, the BBB score was higher than the SCI group after 7days (Fig. 7C).

Nissl staining showed that the color intensity and the number of nissl bodies in the SCI-7d group and the SCI-7d+AAV NC decreased significantly compared with the sham group. Compared with the SCI group, the SCI-7d+AAV Sema3A group showed deeper nissl bodies staining with significantly increased expression of tiger-spotted motor neurons that were smaller than in the sham group (Figs. 7A–7B). These results suggest that suppressing Sema3A/NRP1 signal transduction is beneficial to functional recovery after injury.

## Discussion

In this study, we revealed the related mechanism of Sema3A/NRP1 signaling on OPCs and OLs through bioinformatics analysis. Previous studies (Mctigue, Wei & Stokes, 2001) have shown that OLs are important cells involved in nerve repair after SCI, mainly formed by the proliferation, migration and differentiation of OPCs. Massive OLs death after SCI is accompanied by demyelination which restricts axon electrical signal conduction and leads to dysfunction post-SCI (Totoiu & Keirstead, 2005). Interestingly, cell transplantation can enhance the differentiation of OPCs and OL post-SCI, thus improving myelination and promoting spinal cord nerve function recovery. Our study found that the expression of OL markers olig2 and sox10 decreased significantly post-SCI, indicating that significant OL death post-SCI may seriously hinder the repair of nerve function after injury.

NRP1 was first documented as a key receptor that inhibits the extension of axons when bound to Sema3A (Goshima et al., 2000). Sema3A/NRP1 signaling is one of the key molecular signals for the formation and growth of myelin sheath (Hamashima et al., 2020). Orr, Fetter & Davis (2017) extracted SM-216289, a potent and selective inhibitor of Sema3A, and injected it into the lesion site of rats with spinal cord transection for 4 weeks. The results showed that the rats treated with SM-216289 exhibited axon regeneration, myelination, and angiogenesis, and damaged nerve function was significantly restored. In addition, inhibition of Sema3A/NRP1 signaling can promote motor function recovery after SCI (Zhang et al., 2014). Our previous studies (Hu et al., 2020) confirmed that Sema3A /NRP1 signaling plays an important role in functional recovery after SCI in rats. Herein, we found that the expression of Sema3A and NRP1 was significantly increased after SCI. However, the mechanism of action between Sema3A/NRP1 signal and OLs after SCI is still unclear.

GO and KEGG analyses showed that the most significant upregulated genes were related to wound healing, cell-cytokine interaction, cell proliferation, differentiation and maturation in the SCI group. Meanwhile, NRP1 is well-established to be involved in the semaphorin-plexin signaling pathway neuronal projection guidance and axon guidance, mediating cell growth and migration. Of note, PPI network analysis found that Sema3A/NRP1 signaling may regulate the expression of PDGFR *α*. It is well known that PDGFR *α*, one of the specific markers of OPCs, is produced by both astrocytes and neurons (Hamashima et al., 2020; Duncan et al., 2020). OPCs are tightly correlated with the maturation of OLs and can differentiate into mature OLs (Liu et al., 2017). Hence, we speculated that Sema3A/NRP1 plays an important role in post-injury repair and may be involved in the development of OPCs and OL after SCI.

Evidence has verified that Sema3A is a selective and reversible inhibitor of OPCs differentiation *in vitro* (Syed et al., 2011). Drug inhibition or knockout mice blocking Sema3A/NRP1 signal transduction can increase OPCs recruitment, reduce demyelination lesions (Wang & Li, 2020) and axon growth-inhibitory factor (Boyd, Zhang & Williams, 2013) expression. To clarify the relationship between 3A/NRP1 and OPCs and OLs, we selectively downregulated the expression of NRP1 ligand Sema3A in the spinal cord at the injury site and observed that PDGFR *α* expression was downregulated when the expression of Sema3A/NRP1 signaling was inhibited, while olig2 and sox10 were significantly increased on day 7. These results suggest that inhibition of Sema3A/NRP1 signaling may promote the growth and development of OLs after injury. Moreover, we found that motor function improved with downregulation of Sema3A/NRP1 signaling with increased motor neuron survival in the ventral horn of the spinal cord. According to the above findings, decreasing Sema3A/NRP1 signaling can promote the repair of spinal motor neuron function and lower limb motor function post-SCI.

In conclusion, this study provided compelling evidence that Sema3A/NRP1 signaling can regulate the expression of OPCs and OLs after SCI. Importantly, inhibition of Sema3A/NRP1 signaling can improve the recovery of motor function after SCI.

##  Supplemental Information

10.7717/peerj.13856/supp-1 Figure S1 Schematic of viral injection (a) Schematic diagram of virus injection location (b-d) represents the transfection effect of spinal cord T9, T10 and T11 viruses after injection.

10.7717/peerj.13856/supp-2 Figure S2 PPI network analysis PPI network analysis of the DEGs. Green represents downregulated diûerential genes, red represents upregulated diûerential genes and blue represents key genes. Gray lines represent the protein interaction relationships.

10.7717/peerj.13856/supp-3 Table S1 NRP1 involved in KEGG all NRP1-involved pathways that we summarized from all pathway enrichment results.

10.7717/peerj.13856/supp-4 Figure S2A Gene Ontology

10.7717/peerj.13856/supp-5 Supplemental Information 1 Construction of viral plasmid

10.7717/peerj.13856/supp-6 Supplemental Information 2 ARRIVE checklist

10.7717/peerj.13856/supp-7 Supplemental Information 3 GO analysis R code

10.7717/peerj.13856/supp-8 Supplemental Information 4 KEGG analysis R code

10.7717/peerj.13856/supp-9 Supplemental Information 5 Heatmaps R code

10.7717/peerj.13856/supp-10 Supplemental Information 6 WB original bands

10.7717/peerj.13856/supp-11 Supplemental Information 7 PCR original data

10.7717/peerj.13856/supp-12 Supplemental Information 8 Original data and statistical report of each graph

## Additional Information and Declarations

### Data Availability

The following information was supplied regarding data availability: Raw data is available at NCBI: GSE45006.
