# Supplementary figures and images for: Integrated bioinformatics analysis identifies the effects of Sema3A/NRP1 signaling in oligodendrocytes after spinal cord injury in rats
Source: PeerJ. 2022 Aug 16;10:e13856. doi: 10.7717/peerj.13856 (PMC9390322; doi:10.7717/peerj.13856)

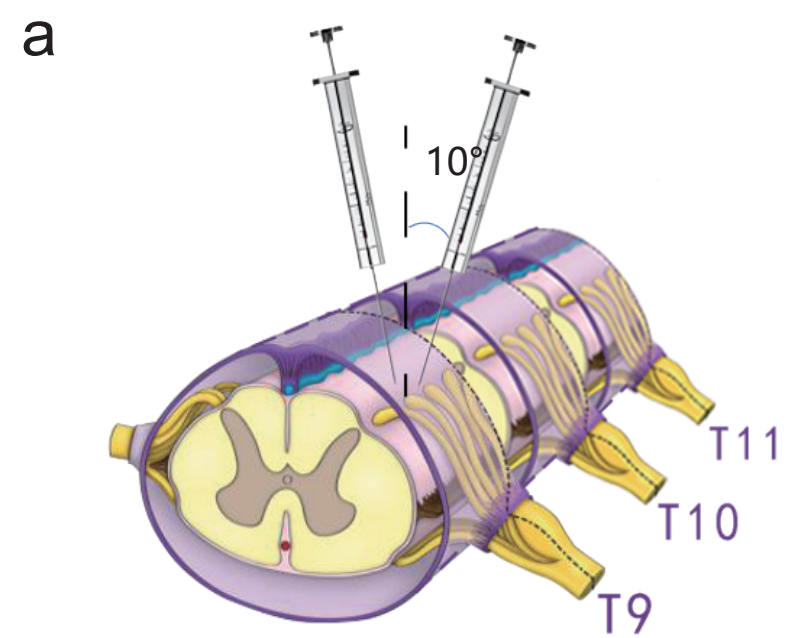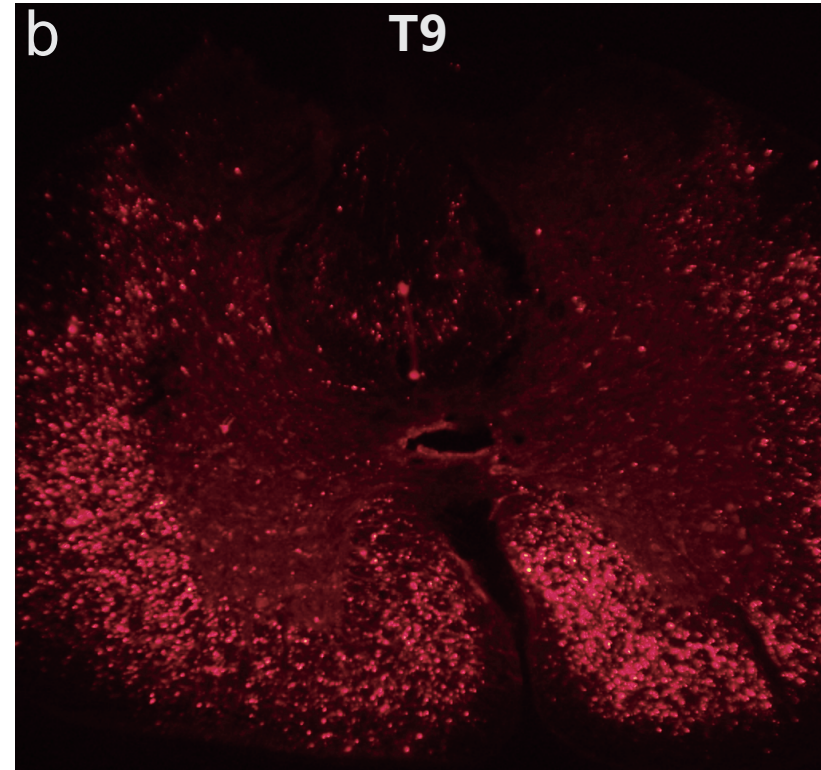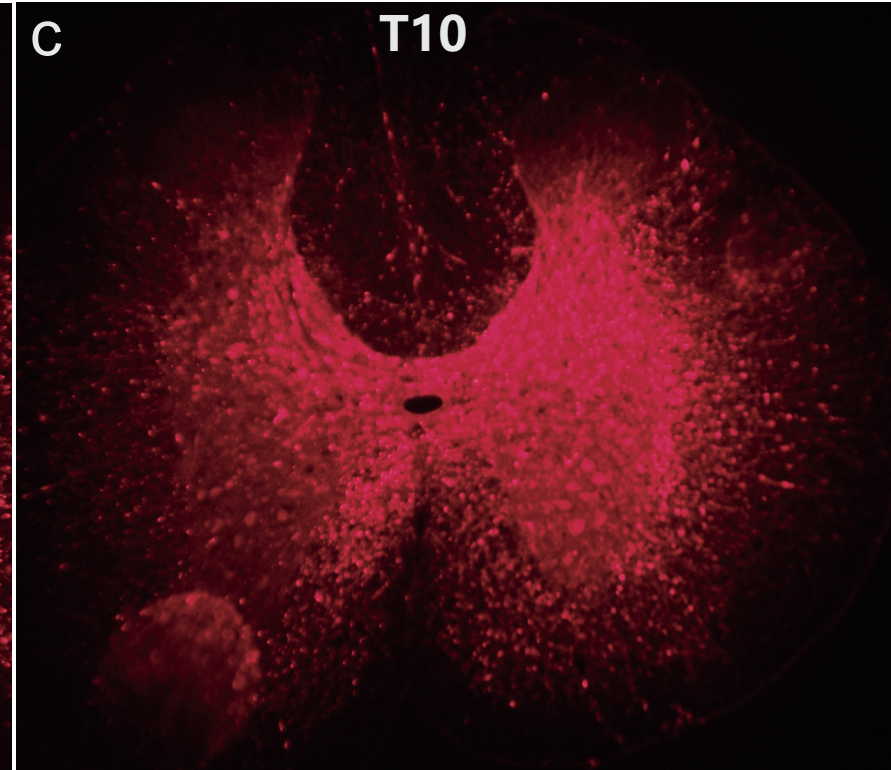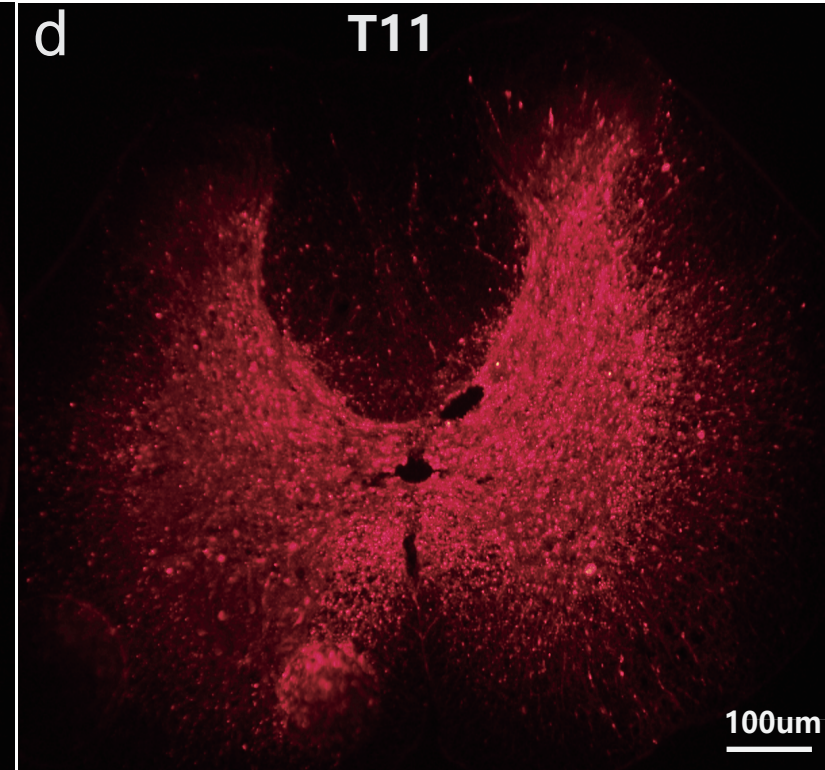

Supplement: Figure S1 — (a) Schematic diagram of virus injection location (b-d) represents the transfection effect of spinal cord T9, T10 and T11 viruses after injection. [file peerj-10-13856-s001.pdf]

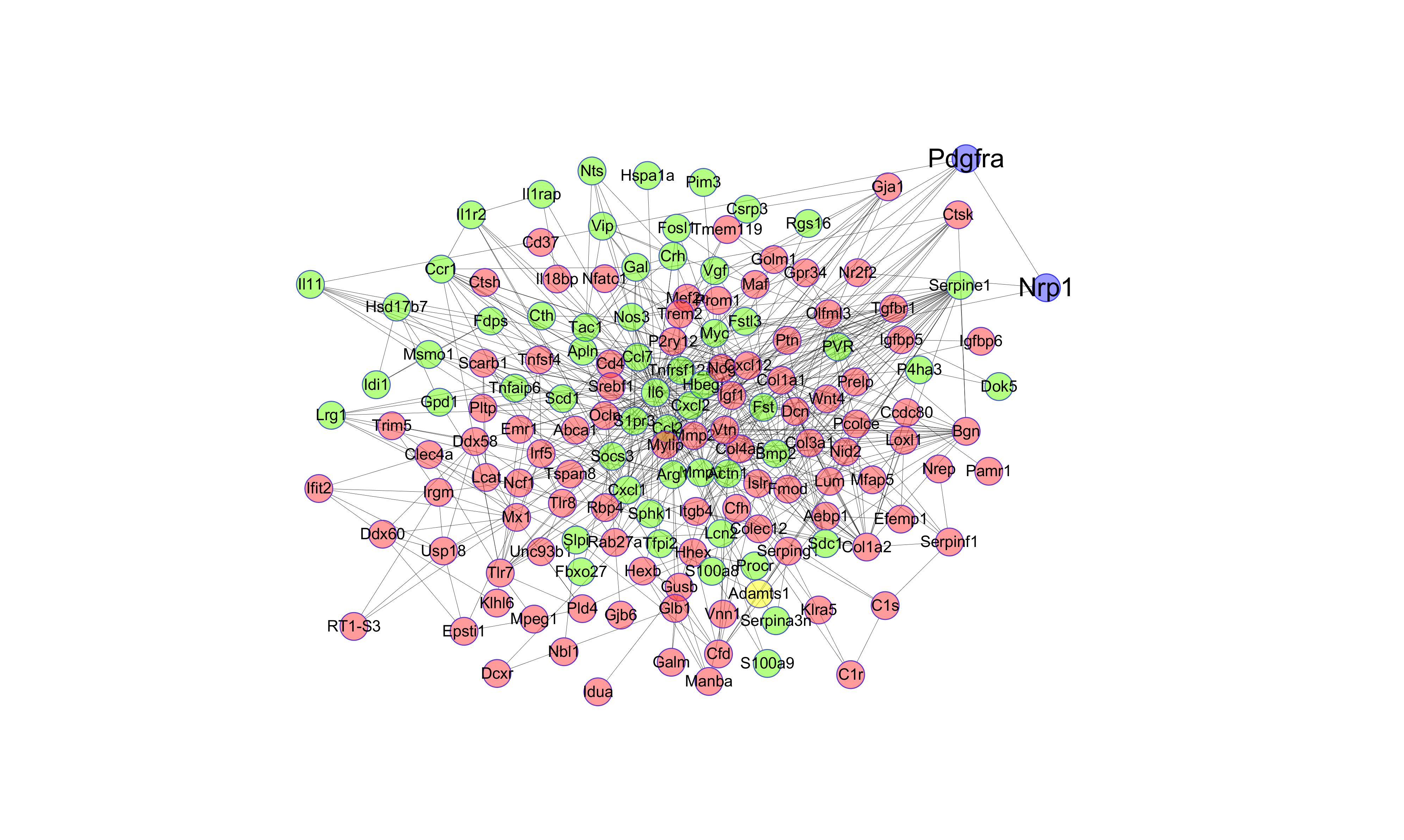

Supplement: Figure S2 — PPI network analysis of the DEGs. Green represents downregulated diûerential genes, red represents upregulated diûerential genes and blue represents key genes. Gray lines represent the protein interaction relationships. [file peerj-10-13856-s002.png]

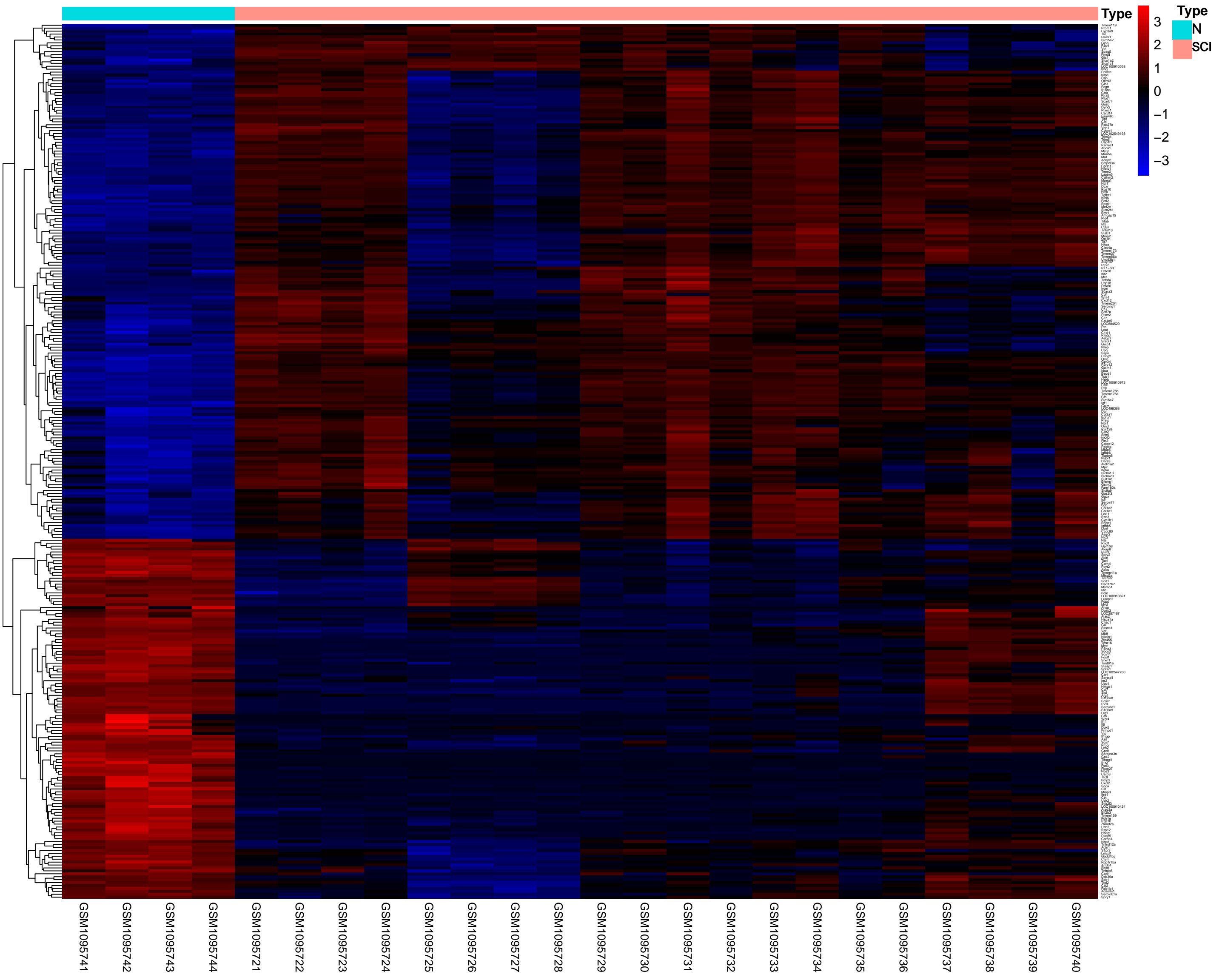

Supplement: Figure S2A [file peerj-10-13856-s004.pdf]

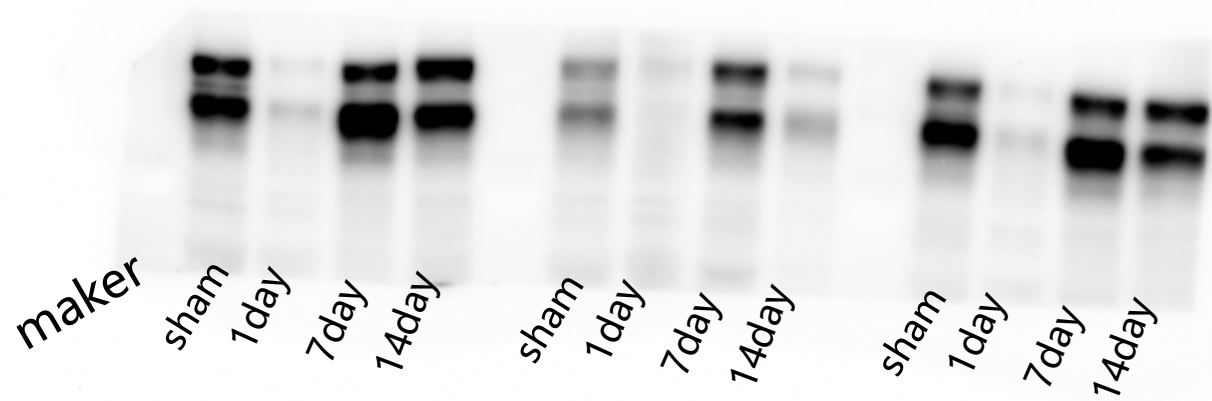

Supplement: Supplemental Information 6 [file peerj-10-13856-s010.zip › WB/20210926_1317_6.pdf]

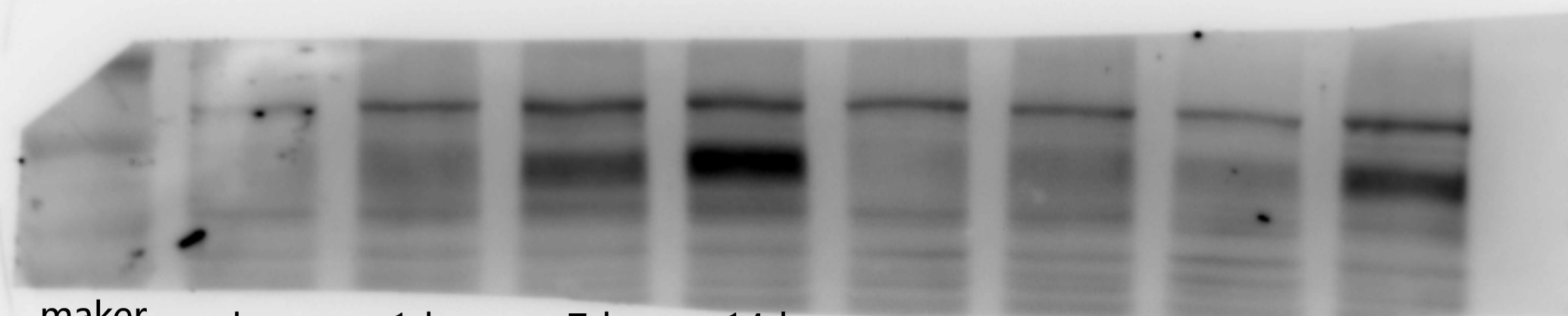

maker

sham

1day

7day

14day

sham

1day

7day

14day

Supplement: Supplemental Information 6 [file peerj-10-13856-s010.zip › WB/20210926_1333_5.pdf]

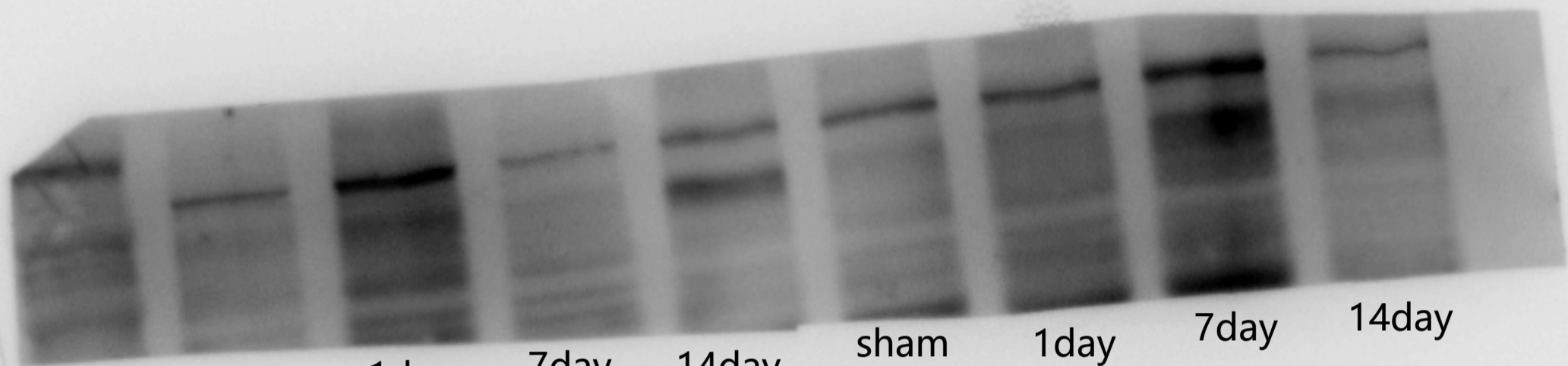

sham

1day

7day

14day

sham

1day

7day

14day

Supplement: Supplemental Information 6 [file peerj-10-13856-s010.zip › WB/20210926_1348 3.pdf]

$\beta$ -actin

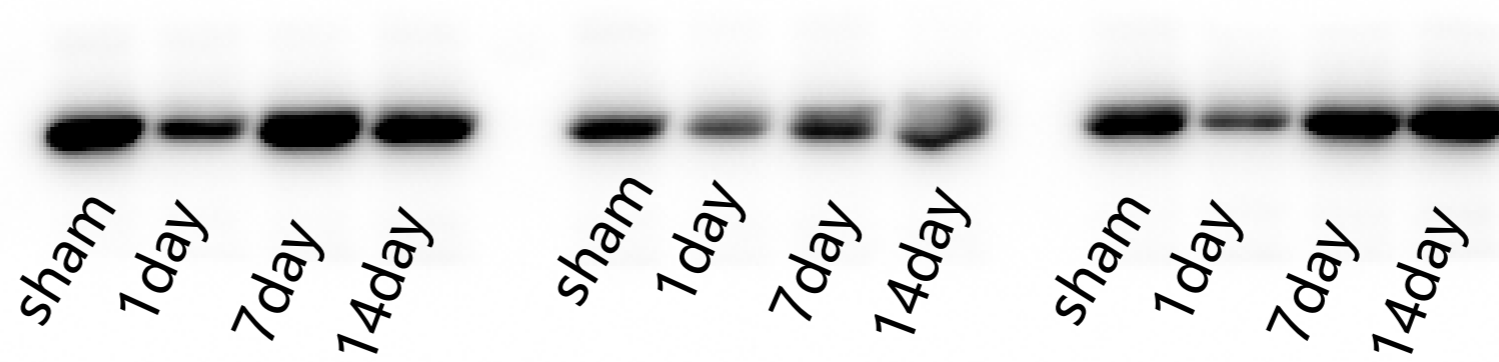

Supplement: Supplemental Information 6 [file peerj-10-13856-s010.zip › WB/20210926_1357.ai 6.pdf]

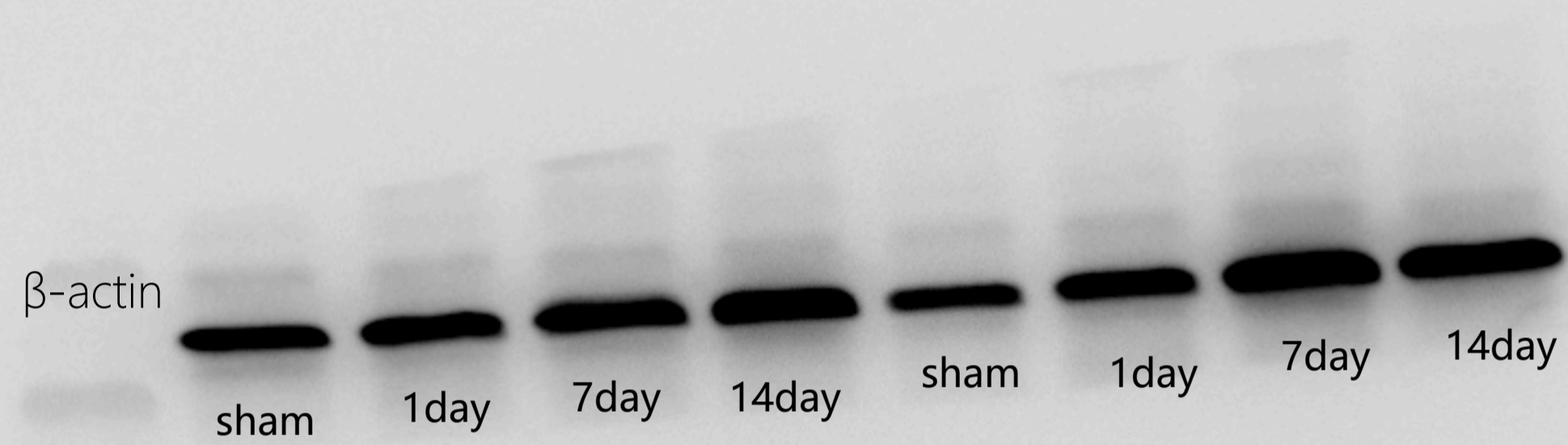

Supplement: Supplemental Information 6 [file peerj-10-13856-s010.zip › WB/20210926_1401.ai 5.pdf]

$\beta$ -actin

sham

1day

7day

14day

sham

1day

7day

14day

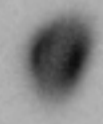

Supplement: Supplemental Information 6 [file peerj-10-13856-s010.zip › WB/20210926_1415_5.ai 3.pdf]

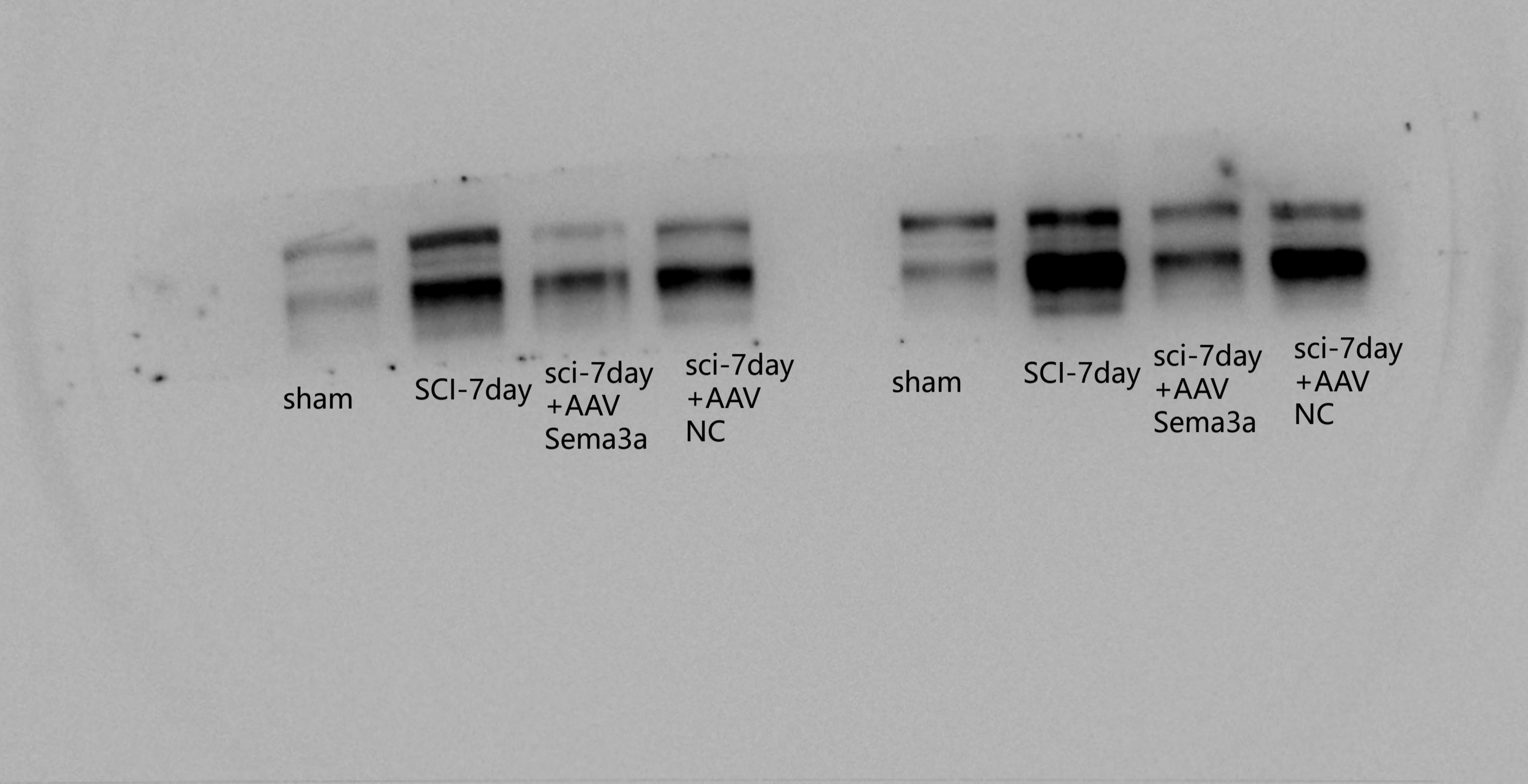

Supplement: Supplemental Information 6 [file peerj-10-13856-s010.zip › WB/20210929_1540_5.pdf]

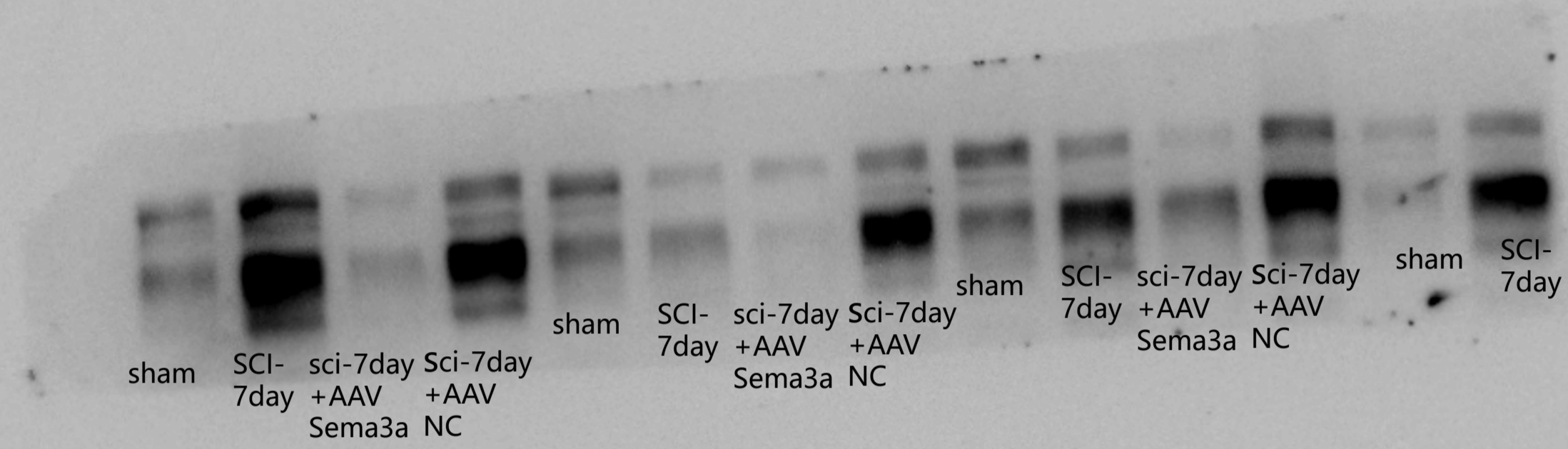

Supplement: Supplemental Information 6 [file peerj-10-13856-s010.zip › WB/20210929_1549_5.pdf]

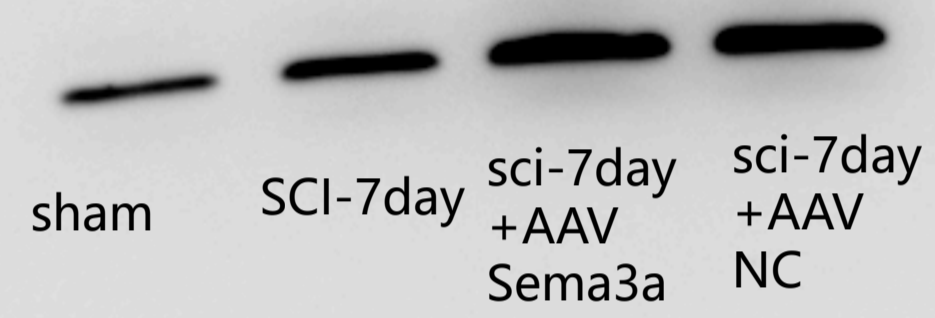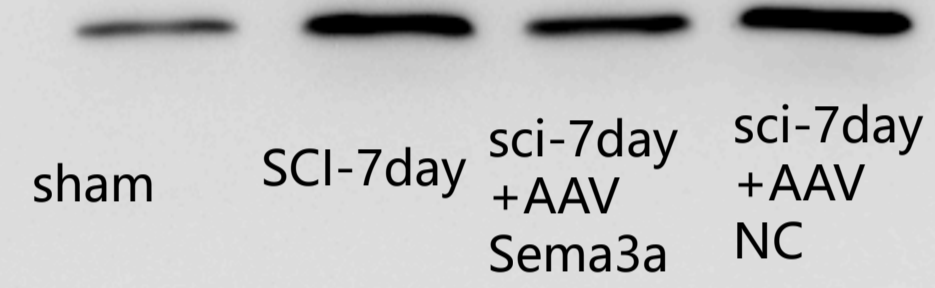

Supplement: Supplemental Information 6 [file peerj-10-13856-s010.zip › WB/20210929_1558_3.pdf]

β-actin

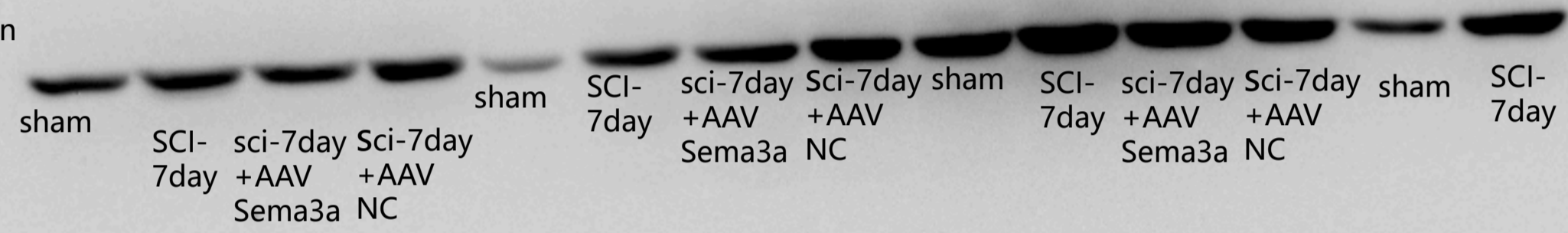

Supplement: Supplemental Information 6 [file peerj-10-13856-s010.zip › WB/20210929_1607_5.pdf]
