# Supplementary material for: Integrated bioinformatics analysis identifies the effects of Sema3A/NRP1 signaling in oligodendrocytes after spinal cord injury in rats
Source: PeerJ. 2022 Aug 16;10:e13856. doi: 10.7717/peerj.13856 (PMC9390322; doi:10.7717/peerj.13856)
Supplement: Supplemental Information 1 [file peerj-10-13856-s005.docx]

Target gene name: Sema3a

Gene Sequence Number: NM_017310.1

Gene: 29751

**Carrier information**

Carrier Name: WZ032

Component order：pAAV-H1-shRNA-CAG-tdTomato-WPRE-SV40pA

Cloning site：BsaI


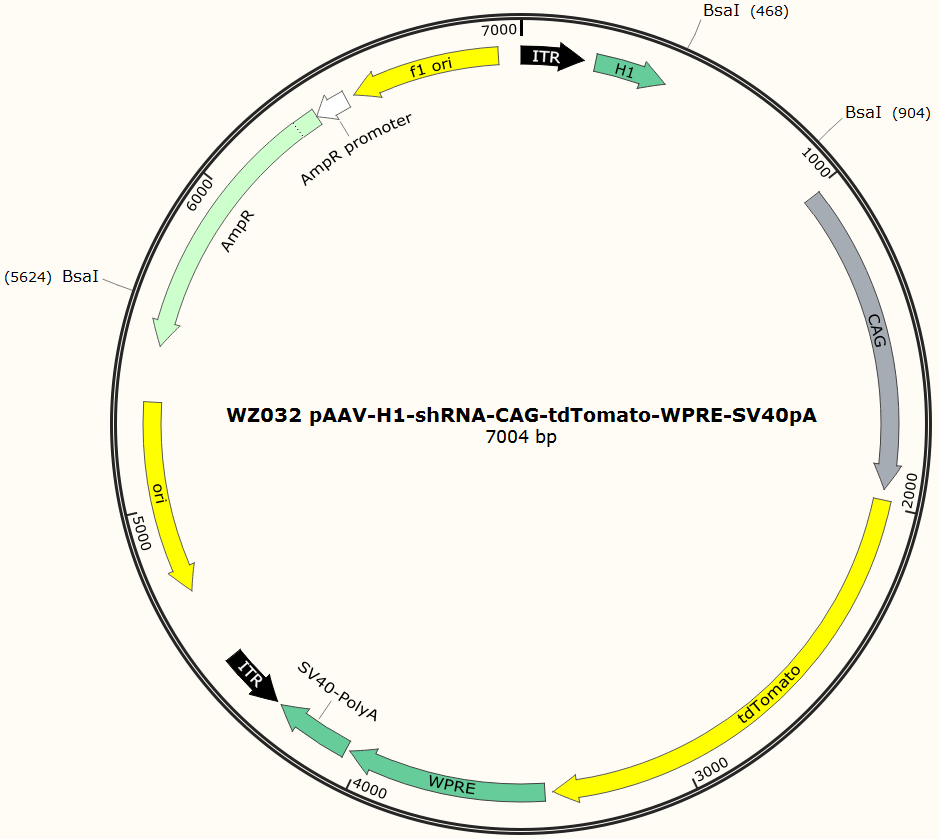


To further verify the impact of Sema3A post SCI, we designed 6 sets of SNCA-shRNA sequence plasmids with GFP fluorescent tags, and integrated them into an adenoassociated virus (AAV), then transfected them into HEK293 cells to observe the degree of translated. We utilize fluorescence attenuation detection co-transfect 293T cells in vitro with the interference group, the interference control sample wy2884-2889, the interference control sample WY1720 and the overexpressed sample WY2890. It was found that the negative control WY1720 of the interference target had no ability to knock-down, while the target sequences of WY2886, WY2887 and WY2889 in the experimental group had a strong knock-down ability (Fig. 5c). Western blotting was further used to check the efficiency of each target and the result displayed WY2887 had the least amount of protein as well as the best interference effect (Fig. 5b). Therefore, we considered wy2887 is the most suitable one among the six sequences of wy2884- 2889. Hence, we chose wy2887 to package AAV2/9-U6-shRNA (Sema3A)- CAG-tdtomato for the following experiment.

Primer sequence

| Y2884-1 | TCCCCGGAAAGAACAATGTGCCAATTCAAGAGATTGGCACATTGTTCTTTCCTTTTT |
| --- | --- |
| Y2884-2 | TCTAAAAAAGGAAAGAACAATGTGCCAATCTCTTGAATTGGCACATTGTTCTTTCCG |
|  |  |
| Y2885-1 | TCCCCGCCATCCAATTTGCACCTATTTCAAGAGAATAGGTGCAAATTGGATGGCTTTTT |
| Y2885-2 | TCTAAAAAAGCCATCCAATTTGCACCTATTCTCTTGAAATAGGTGCAAATTGGATGGCG |
|  |  |
| Y2886-1 | TCCCCGCCTGAAGATGACAAAGTATTTCAAGAGAATACTTTGTCATCTTCAGGCTTTTT |
| Y2886-2 | TCTAAAAAAGCCTGAAGATGACAAAGTATTCTCTTGAAATACTTTGTCATCTTCAGGCG |
|  |  |
| Y2887-1 | TCCCCGCTAGAATAGGTCAGATATTTCAAGAGAATATCTGACCTATTCTAGCTTTTT |
| Y2887-2 | TCTAAAAAAGCTAGAATAGGTCAGATATTCTCTTGAAATATCTGACCTATTCTAGCG |
|  |  |
| Y2888-1 | TCCCCGCAATGGAGCTTTCTACTATTCAAGAGATAGTAGAAAGCTCCATTGCTTTTT |
| Y2888-2 | TCTAAAAAAGCAATGGAGCTTTCTACTATCTCTTGAATAGTAGAAAGCTCCATTGCG |
|  |  |
| Y2889-1 | TCCCCGGATGAGTTCTGTGAACAATTCAAGAGATTGTTCACAGAACTCATCCTTTTT |
| Y2889-2 | TCTAAAAAAGGATGAGTTCTGTGAACAATCTCTTGAATTGTTCACAGAACTCATCCG |
