# Supplementary material for: Integrated bioinformatics analysis identifies the effects of Sema3A/NRP1 signaling in oligodendrocytes after spinal cord injury in rats
Source: PeerJ. 2022 Aug 16;10:e13856. doi: 10.7717/peerj.13856 (PMC9390322; doi:10.7717/peerj.13856)
Supplement: Supplemental Information 8 [file peerj-10-13856-s012.zip › Original data and statistical report of each graph/BBB .pdf]

BBB score

	SCI-7d+AAV Sema3A	SCI-7d+AAV	sham	SCI-7d
0	21	21	21	21
1	0.333333	0.454546	21	0.181818
3	1.583333	0.636364	21	0.363636
5	3.909091	1.7	21	0.5
7	7.636364	2.4	21	1.833333

	SCI-7d+AAV Sema3A	SCI-7d+AAV	sham	SCI-7d
Number of valu	5	5	5	5
Minimum	0.3333	0.4545	21	0.1818
Maximum	21	21	21	21
Range	20.67	20.55	0	20.82
10% Percentile	0.3333	0.4545	21	0.1818
90% Percentile	21	21	21	21
Mean	6.892	5.238	21	4.776
Std. Deviation	8.362	8.847	0	9.093
Std. Error of Me	3.74	3.956	0	4.067
