# Supplementary material for: Integrated bioinformatics analysis identifies the effects of Sema3A/NRP1 signaling in oligodendrocytes after spinal cord injury in rats
Source: PeerJ. 2022 Aug 16;10:e13856. doi: 10.7717/peerj.13856 (PMC9390322; doi:10.7717/peerj.13856)
Supplement: Supplemental Information 8 [file peerj-10-13856-s012.zip › Original data and statistical report of each graph/figure1.pdf]

figure1 a

| | | | | |
|---|---|---|---|---|
| Number of valu | 3 | 9 | 4 | 8 |
| | | | | |
| Minimum | 0.8636 | 0.02441 | 0.06222 | 0.01403 |
| Maximum | 1.177 | 0.1446 | 0.1514 | 0.1446 |
| Range | 0.3134 | 0.1202 | 0.08922 | 0.1306 |
| | | | | |
| 95% CI of median | | | | |
| Actual confiden | 75.00% | 96.09% | 87.50% | 99.22% |
| Lower confiden | 0.8636 | 0.02515 | 0.06222 | 0.01403 |
| Upper confiden | 1.177 | 0.06397 | 0.1514 | 0.1446 |
| | | | | |
| Mean | 1 | 0.04943 | 0.09632 | 0.07049 |
| Std. Deviation | 0.1606 | 0.03789 | 0.04014 | 0.04079 |
| Std. Error of Me | 0.09272 | 0.01263 | 0.02007 | 0.01442 |

figure1 b

| | sham | 1day | 7day | 14day |
|---|---|---|---|---|
| Number of valu | 8 | 7 | 5 | 5 |
| | | | | |
| Minimum | 0.7943 | 0.08378 | 0.1297 | 0.04112 |
| Maximum | 1.257 | 0.7302 | 0.4336 | 0.5507 |
| Range | 0.4624 | 0.6464 | 0.3039 | 0.5096 |
| | | | | |
| 95% CI of median | | | | |
| Actual confiden | 99.22% | 98.44% | 93.75% | 93.75% |
| Lower confiden | 0.7943 | 0.08378 | 0.1297 | 0.04112 |
| Upper confiden | 1.257 | 0.7302 | 0.4336 | 0.5507 |
| | | | | |
| Mean | 1.005 | 0.4535 | 0.2448 | 0.2519 |
| Std. Deviation | 0.1947 | 0.1939 | 0.1172 | 0.19 |
| Std. Error of Me | 0.06884 | 0.07327 | 0.05239 | 0.08498 |

figure1 c

| | sham | 1day | 7day | 14day |
|---|---|---|---|---|
| Number of valu | 9 | 7 | 4 | 3 |
| | | | | |
| Minimum | 0.5245 | 0.2126 | 0.7934 | 3.521 |
| Maximum | 1.135 | 0.5779 | 2.323 | 5.084 |
| Range | 0.6106 | 0.3653 | 1.53 | 1.563 |
| | | | | |
| 95% CI of median | | | | |
| Actual confiden | 96.09% | 98.44% | 87.50% | 75.00% |
| Lower confiden | 0.6893 | 0.2126 | 0.7934 | 3.521 |
| Upper confiden | 1.109 | 0.5779 | 2.323 | 5.084 |
| | | | | |
| Mean | 0.8661 | 0.3837 | 1.637 | 4.536 |
| Std. Deviation | 0.2076 | 0.1338 | 0.6315 | 0.8799 |
| Std. Error of Me | 0.06919 | 0.05058 | 0.3158 | 0.508 |

figure1 d

| | sham | 1day | 7day | 14day |
|---|---|---|---|---|
| Number of valu | 4 | 7 | 7 | 10 |
| | | | | |
| Minimum | 0.4794 | 0.7304 | 0.6747 | 0.6789 |
| Maximum | 0.705 | 1.306 | 1.123 | 1.6 |
| Range | 0.2256 | 0.5761 | 0.4487 | 0.9211 |

| | | | | |
|---|---|---|---|---|
| 95% CI of median | | | | |
| Actual confiden | 87.50% | 98.44% | 98.44% | 97.85% |
| Lower confiden | 0.4794 | 0.7304 | 0.6747 | 0.7225 |
| Upper confiden | 0.705 | 1.306 | 1.123 | 1.306 |
| | | | | |
| Mean | 0.598 | 0.9615 | 0.8789 | 0.9783 |
| Std. Deviation | 0.1154 | 0.1788 | 0.1416 | 0.2866 |
| Std. Error of Me | 0.05769 | 0.06756 | 0.05352 | 0.09062 |
