# Supplementary material for: Integrated bioinformatics analysis identifies the effects of Sema3A/NRP1 signaling in oligodendrocytes after spinal cord injury in rats
Source: PeerJ. 2022 Aug 16;10:e13856. doi: 10.7717/peerj.13856 (PMC9390322; doi:10.7717/peerj.13856)
Supplement: Supplemental Information 8 [file peerj-10-13856-s012.zip › Original data and statistical report of each graph/figure2d.pdf]

figure2d-left

Number of values	3	5
Minimum	0.7743	2.554
Maximum	1.073	3.743
Range	0.2982	1.189
95% CI of median		
Actual confidence level	75.00%	93.75%
Lower confidence limit	0.7743	2.554
Upper confidence limit	1.073	3.743
Mean	0.9226	3.26
Std. Deviation	0.1491	0.4384
Std. Error of Mean	0.08609	0.1961

figure2d-right

Number of values	5	5
Minimum	0.8074	1.361
Maximum	1.16	1.916
Range	0.353	0.5549
95% CI of median		
Actual confidence level	93.75%	93.75%
Lower confidence limit	0.8074	1.361
Upper confidence limit	1.16	1.916
Mean	1.008	1.64
Std. Deviation	0.1372	0.2571
Std. Error of Mean	0.06135	0.115
Std. Error of Mean	0.06135	0.115
