# Supplementary material for: Integrated bioinformatics analysis identifies the effects of Sema3A/NRP1 signaling in oligodendrocytes after spinal cord injury in rats
Source: PeerJ. 2022 Aug 16;10:e13856. doi: 10.7717/peerj.13856 (PMC9390322; doi:10.7717/peerj.13856)
Supplement: Supplemental Information 8 [file peerj-10-13856-s012.zip › Original data and statistical report of each graph/figure5.pdf]

figure5b

	sham	SCI-7d	SCI-7d+AAV Sema3A	SCI-7d+AAV NC
Number of values	3	3	3	4
Minimum	4.333	45	14	45
Maximum	8.333	65.33	19.67	62
Range	4	20.33	5.667	17
95% CI of median				
Actual confidence level	75.00%	75.00%	75.00%	87.50%
Lower confidence limit	4.333	45	14	45
Upper confidence limit	8.333	65.33	19.67	62
Mean	6.111	56.67	17.11	54.17
Std. Deviation	2.037	10.49	2.874	7.356
Std. Error of Mean	1.176	6.058	1.659	3.678

figure5c

	sham	SCI-7d	SCI-7d+AAV Sema3A	SCI-7d+AAV NC
Number of values	3	3	3	3
Minimum	17	94	36.67	106.7
Maximum	21.33	132.7	44.67	121
Range	4.333	38.67	8	14.33
95% CI of median				
Actual confidence level	75.00%	75.00%	75.00%	75.00%
Lower confidence limit	17	94	36.67	106.7
Upper confidence limit	21.33	132.7	44.67	121
Mean	19.78	110	40.78	115.2
Std. Deviation	2.411	20.18	4.005	7.56
Std. Error of Mean	1.392	11.65	2.312	4.365

figure5d

	sham	SCI-7d	SCI-7d+AAV Sema3A	SCI-7d+AAV NC
Number of values	5	3	4	5
Minimum	0.6103	3.312	0.7851	1.438
Maximum	1.622	3.743	1.889	3.327
Range	1.012	0.4315	1.104	1.889
95% CI of median				
Actual confidence level	93.75%	75.00%	87.50%	93.75%
Lower confidence limit	0.6103	3.312	0.7851	1.438
Upper confidence limit	1.622	3.743	1.889	3.327
Mean	1	3.5	1.168	1.944
Std. Deviation	0.3876	0.221	0.4995	0.7864
Std. Error of Mean	0.1734	0.1276	0.2498	0.3517

figure5e

Number of values	6	6	6	5
Minimum	0.7501	1.895	1.119	1.839
Maximum	1.532	3.106	1.764	3.407

Range	0.7817	1.212	0.6449	1.569
95% CI of median				
Actual confidence level	96.88%	96.88%	96.88%	93.75%
Lower confidence limit	0.7501	1.895	1.119	1.839
Upper confidence limit	1.532	3.106	1.764	3.407
Mean	1	2.42	1.433	2.51
Std. Deviation	0.2837	0.4595	0.2895	0.5817
Std. Error of Mean	0.1158	0.1876	0.1182	0.2602
