# Supplementary material for: Integrated bioinformatics analysis identifies the effects of Sema3A/NRP1 signaling in oligodendrocytes after spinal cord injury in rats
Source: PeerJ. 2022 Aug 16;10:e13856. doi: 10.7717/peerj.13856 (PMC9390322; doi:10.7717/peerj.13856)
Supplement: Supplemental Information 8 [file peerj-10-13856-s012.zip › Original data and statistical report of each graph/figure7.pdf]

figure7a

	sham	SCI-7d	SCI-7d+AAV Sema3A	SCI-7d+AAV NC
Minimum	0.8636	0.04946	0.6654	0.06222
Maximum	1.177	0.4211	0.9027	0.1962
Range	0.3134	0.3717	0.2373	0.1339
95% CI of median				
Actual confidence level	75.00%	93.75%	75.00%	93.75%
Lower confidence limit	0.8636	0.04946	0.6654	0.06222
Upper confidence limit	1.177	0.4211	0.9027	0.1962
Mean	1	0.1696	0.7908	0.1163
Std. Deviation	0.1606	0.1454	0.1192	0.05659
Std. Error of Mean	0.09272	0.06501	0.06883	0.02531

figure7b

	sham	SCI-7d	SCI-7d+AAV Sema3A	SCI-7d+AAV NC
Minimum	0.935	0.2918	0.7003	0.4998
Maximum	1.257	0.6183	0.8056	0.559
Range	0.3217	0.3265	0.1052	0.05918
95% CI of median				
Actual confidence level	87.50%	93.75%	75.00%	75.00%
Lower confidence limit	0.935	0.2918	0.7003	0.4998
Upper confidence limit	1.257	0.6183	0.8056	0.559
Mean	1.095	0.4463	0.7617	0.5337
Std. Deviation	0.136	0.1165	0.05476	0.03055
Std. Error of Mean	0.06798	0.05209	0.03161	0.01764

figure7c

	sham	SCI-7d	SCI-7d+AAV Sema3A	SCI-7d+AAV NC
Minimum	0.6228	1.248	0.1205	1.257
Maximum	0.9527	2.323	1.295	2.334
Range	0.3299	1.075	1.175	1.078
95% CI of median				
Actual confidence level	99.22%	96.88%	98.83%	93.75%
Lower confidence limit	0.6228	1.248	0.1278	1.257
Upper confidence limit	0.9527	2.323	1.089	2.334
Mean	0.7689	1.739	0.5113	1.858
Std. Deviation	0.1045	0.3427	0.4285	0.4646
Std. Error of Mean	0.03694	0.1399	0.1292	0.2078

figure7d

	sham	SCI-7d	SCI-7d+AAV Sema3A	SCI-7d+AAV NC
Minimum	0.2696	0.6031	0.08335	0.8406
Maximum	1.391	1.655	0.7811	1.151
Range	1.121	1.052	0.6977	0.3107
95% CI of median				
Actual confidence level	98.83%	98.83%	98.83%	96.88%
Lower confidence limit	0.3233	0.6747	0.2384	0.8406
Upper confidence limit	0.9097	1.244	0.6507	1.151
Mean	0.5839	1.049	0.4441	0.996

Std. Deviation	0.3346	0.303	0.2073	0.1133
Std. Error of Mean	0.1009	0.09136	0.0625	0.04627

figure7e

	sham	SCI-14d	SCI-14d+AAV Sema3A	SCI-14d+AAV NC
Minimum	0.6289	0.0278	0.002882	0.01403
Maximum	1.177	0.1334	0.0831	0.2493
Range	0.5481	0.1056	0.08022	0.2353
95% CI of median				
Actual confidence level	93.75%	96.88%	97.85%	99.22%
Lower confidence limit	0.6289	0.0278	0.01578	0.01403
Upper confidence limit	1.177	0.1334	0.07559	0.2493
Mean	0.8563	0.07977	0.04888	0.07295
Std. Deviation	0.2273	0.03954	0.02661	0.07883
Std. Error of Mean	0.1017	0.01614	0.008415	0.02787

figure7f

	sham	SCI-14d	SCI-14d+AAV Sema3A	SCI-14d+AAV NC
Minimum	0.5074	0.1713	0.2148	0.2729
Maximum	1.177	0.3035	0.5097	0.5572
Range	0.6697	0.1322	0.2949	0.2843
95% CI of median				
Actual confidence level	96.88%	96.88%	93.75%	96.88%
Lower confidence limit	0.5074	0.1713	0.2148	0.2729
Upper confidence limit	1.177	0.3035	0.5097	0.5572
Mean	0.9026	0.2211	0.355	0.4159
Std. Deviation	0.2231	0.04455	0.1361	0.1223
Std. Error of Mean	0.09109	0.01819	0.06088	0.04995

figure7g

	sham	SCI-14d	SCI-14d+AAV Sema3A	SCI-14d+AAV NC
Minimum	0.6228	2.234	0.1161	1.903
Maximum	1.135	3.882	1.903	3.521
Range	0.5122	1.648	1.787	1.618
95% CI of median				
Actual confidence level	98.83%	93.75%	96.09%	87.50%
Lower confidence limit	0.7047	2.234	0.1297	1.903
Upper confidence limit	1.109	3.882	0.4152	3.521
Mean	0.9022	3.095	0.4242	2.495
Std. Deviation	0.1628	0.7627	0.5631	0.7074
Std. Error of Mean	0.0491	0.3411	0.1877	0.3537
