# Supplementary material for: Integrated bioinformatics analysis identifies the effects of Sema3A/NRP1 signaling in oligodendrocytes after spinal cord injury in rats
Source: PeerJ. 2022 Aug 16;10:e13856. doi: 10.7717/peerj.13856 (PMC9390322; doi:10.7717/peerj.13856)
Supplement: Supplemental Information 8 [file peerj-10-13856-s012.zip › Original data and statistical report of each graph/figure8 b c.pdf]

figure8 b

	normal motor neuron index			
	sham	M	M+AAV NC	M+AAV Sema3A
	21	1.66666	6.33333	9
normal motor neuron	23.33	3.33	5.333333	10
	25	5.666667	3.666667	15.33333
Number of values	3	3	3	3
Minimum	21	1.667	3.667	9
Maximum	25	5.667	6.333	15.33
Range	4	4	2.667	6.333
10% Percentile	21	1.667	3.667	9
90% Percentile	25	5.667	6.333	15.33
Mean	23.11	3.554	5.111	11.44
Std. Deviation	2.009	2.009	1.347	3.405
Std. Error of Mear	1.16	1.16	0.7778	1.966

figure8 c

	BBB score			
	SCI-7d+AAV	SCI-7d+AAV	sham	SCI-7d
0	21	21	21	21
1	0.333333	0.454546	21	0.181818
3	1.583333	0.636364	21	0.363636
5	3.909091	1.7	21	0.5
7	7.636364	2.4	21	1.833333

	SCI-7d+AAV	SCI-7d+AAV	sham	SCI-7d
Number of values	5	5	5	5
Minimum	0.3333	0.4545	21	0.1818
Maximum	21	21	21	21
Range	20.67	20.55	0	20.82
10% Percentile	0.3333	0.4545	21	0.1818
90% Percentile	21	21	21	21
Mean	6.892	5.238	21	4.776
Std. Deviation	8.362	8.847	0	9.093
Std. Error of Mear	3.74	3.956	0	4.067
