# Supplementary material for: Integrated bioinformatics analysis identifies the effects of Sema3A/NRP1 signaling in oligodendrocytes after spinal cord injury in rats
Source: PeerJ. 2022 Aug 16;10:e13856. doi: 10.7717/peerj.13856 (PMC9390322; doi:10.7717/peerj.13856)
Supplement: Supplemental Information 8 [file peerj-10-13856-s012.zip › Original data and statistical report of each graph/nissl .pdf]

| | normal motor neuron index | | | |
|---|---|---|---|---|
| | sham | M | M+AAV NC | M+AAV Sema3A |
| normal motor neuron | 21 | 1.66666 | 6.33333 | 9 |
| | 23.33 | 3.33 | 5.333333 | 10 |
| | 25 | 5.666667 | 3.666667 | 15.33333 |
| Number of values | 3 | 3 | 3 | 3 |
| Minimum | 21 | 1.667 | 3.667 | 9 |
| Maximum | 25 | 5.667 | 6.333 | 15.33 |
| Range | 4 | 4 | 2.667 | 6.333 |
| 10% Percentile | 21 | 1.667 | 3.667 | 9 |
| 90% Percentile | 25 | 5.667 | 6.333 | 15.33 |
| Mean | 23.11 | 3.554 | 5.111 | 11.44 |
| Std. Deviation | 2.009 | 2.009 | 1.347 | 3.405 |
| Std. Error of Mear | 1.16 | 1.16 | 0.7778 | 1.966 |
